# A Pharmacy Student–Facilitated Interprofessional Diabetes Clinic With the Penobscot Nation

**DOI:** 10.5888/pcd12.150295

**Published:** 2015-11-05

**Authors:** Sarah Levin Martin, Evan Williams, Benjamin Huerth, J. Daniel Robinson, for the Penobscot Nation Health Center’s Diabetes Team

**Affiliations:** Author Affiliations: Evan Williams, J. Daniel Robinson, Husson University School of Pharmacy, Bangor, Maine; Benjamin Huerth, Penobscot Nation Health Center, Indian Island, Maine.

## Abstract

**Background:**

American Indians/Alaska Natives have a greater increased risk for diabetes than non-Hispanic whites. Lifestyle interventions are effective in preventing and treating diabetes, and an interprofessional approach is important in diabetes management.

**Community Context:**

The Penobscot Nation has a health center with a wide range of services. Our goal with the Nation was to 1) establish an interprofessional, student-facilitated diabetes clinic in the health center; 2) assess the clinic’s preliminary impact.

**Methods:**

Relationship building and problem solving was instrumental in working toward the first goal. A survey was developed to assess satisfaction with the clinic. The clinical outcomes, mean and median values of HbA1c, were calculated at baseline (spring 2013) and were used to establish 2 groups of patients: those with controlled levels (<7%) and those with uncontrolled levels (≥ 7%). HbA1c was reassessed in fall 2013. Changes in HbA1c were calculated and compared using the Wilcoxon signed-rank test.

**Outcomes:**

The student-facilitated, interprofessional diabetes clinic has operated for 2 years, and changes are under way. More than 90% of participants reported being well satisfied with the clinic in the first year. Among the group with uncontrolled HbA1c (n = 18), mean HbA1c values declined from 9.3% to 7.6% (*P* = .004). Among the group with controlled HbA1c (n = 30), 83% were controlled at follow-up.

**Interpretation:**

The Penobscot diabetes clinic is evolving to meet the needs of community members, and pharmacy students have an interprofessional practice site well suited for experiential learning.

## Background

American Indian and Alaska Native (AI/AN) people have a disproportionate share of the diabetes burden. From 2000 to 2009, the age-adjusted diabetes mortality rate for AI/AN adults was 2.5 to 3.5 as times as high as the rate for non-Hispanic white adults; in 2009, the rate was 89.6 per 100,000 for AI/AN adults and 25.0 per 100,000 for white adults (1). Although diabetes among the Pima Indians, who live in an area consisting of what is now central and southern Arizona, is well-studied (2,3), less is known about diabetes among AI/AN people in the northeastern United States. The Wabanaki include the 4 Indian tribes in Maine: Maliseet, Micmac, Passamaquoddy, and Penobscot (4). Diabetes research is lacking for these AI/AN people; the best estimates of diabetes prevalence are based on data from the eastern region of Indian Health Service (IHS), known to be the most geographically and culturally diverse area in IHS (5). In this region, the diabetes mortality rate increased from 59.2% in 1990 to 68.3% in 2009 (1).

The high cost of treating and managing diabetes and its complications increases the overall burden of the disease (6).The development and adoption of a comprehensive approach to diabetes prevention, treatment, and management, including an interdisciplinary health care team, is a high priority for AI/AN communities. Pharmacists can play a key role on the health care team. Interventions for adults with diabetes that include a role for pharmacists can result in reductions of HbA1c, thereby reducing long-term costs and preventing complications (7). Herein, we report on the expansion of a team-based approach to include pharmacy students from a nearby university to provide diabetes care for members of the Penobscot Nation.

## Community Context

Today, more than 1,000 Penobscot people live in Maine, most on or within a 50-mile radius of the reservation (8). Historically, the Penobscot people thrived on a diet and lifestyle reliant on the Penobscot River, including fishing for fresh trout and salmon. Over time, dams, industrial pollution, and societal injustice contributed to increasing levels of ill health. Penobscot tribal members have high rates of poverty and unemployment that exceed state averages. The strength of the Penobscot people is their sense of community, solidarity, shared values, and cultural identity (8).

In 2012, a health needs assessment was conducted through a face-to-face survey among the Penobscot people (8). When asked to indicate the 5 biggest health problems in their community, survey respondents mentioned diabetes more than they did any other problem. According to the needs assessment, which included data abstracted from medical records, diabetes prevalence was 19%, compared with 10% in the state as measured by the Behavioral Risk Factor Surveillance System (8). A more recent chart review at the health center in 2013 showed 174 diagnosed cases of diabetes among the client population of about 1,787, or 11% of the health center’s patients (personal communication, A. McCarthy, nutritionist, Penobscot Nation Health Center’s Diabetes Team, April 20, 2015).

The health center’s administration (clinical director and medical director) wanted to establish a protocol for diabetes management because it was a primary concern to the community at large and the medical community. To begin, they formed a diabetes team in 2012, consisting of the in-house physician, nurse practitioners, nurses, pharmacists, and a registered dietitian. Team members worked collaboratively during the year to devise a plan to best address the needs of patients with diabetes. By year-end, in conjunction with the Husson University School of Pharmacy, they developed the following 2 objectives: 1) to establish an interprofessional, student-facilitated diabetes clinic in the health center, and 2) to monitor the preliminary impact of the clinic on its patients.

## Methods

### Establishment of the diabetes clinic

Although the health center had already been providing comprehensive diabetes care, a formal protocol for care was not in place, and the role of pharmacy was limited. In 2012, a professor of pharmacy practice (J.D.R.) at Husson University met with health center administrators and staff to discuss the creation of an experiential practice site for pharmacy students. Standard formal agreements between the university and the health center were established to ensure legal protection for both parties. These agreements outlined the privileges and restrictions under which university faculty members and the students could operate at the health center and determined parameters on the use of space and shared resources for educational purposes.

Monthly meetings were held with the common goal of creating an interprofessional diabetes clinic to include pharmacists and pharmacy students. During these meetings, roadblocks were often a focus: the primary hurdles were allocation of staff time to schedule patients and various other logistical considerations, such as arranging laboratory tests before each clinic visit and apportioning the time of health care providers. Another area of concern was one of trust: was the university involvement to be dependable and long-term? Continued assurance from the university faculty member (J.D.R.) to provide monthly clinics facilitated by pharmacy students during full-time clinical practice experiences for 42 weeks of the year with him and the Penobscot Health Department Director of Pharmacy was enough of a guarantee to begin the program. 

The students on site were provided cultural sensitivity training in their didactic curriculum, and before they engaged with health center patients, they discussed the special considerations of the AI/AN population with the university faculty member. 

### Diabetes clinic protocol

The newly established protocol for managing patients with diabetes created by the diabetes team called for the addition of pharmacy students and their university and onsite pharmacists as preceptors and was approved by the Health Director for the Tribe in January 2013. The clinic was held during 1 designated day each month, except for December and January. The first clinic took place in spring 2013. The first step in the protocol was that pharmacy students called each health center patient with a diabetes diagnosis to schedule an appointment with the clinic. Through a chart audit, students identified potential issues with the pharmacotherapy regimens of the patients and ensured that laboratory tests were obtained before each visit. The students also provided reminder telephone calls 1 week before the appointment and 1 day before the appointment. Before each designated clinic day, students presented information on the scheduled patients to their preceptor for approval of any recommended changes in medication therapy.

The clinic protocol states that at each visit, each patient first meets with a nurse, who measures the patient’s weight and blood pressure, provides any immunizations that are needed, and documents any clinical issues that need to be addressed during the patient visit. The patient then meets with pharmacy students; under the supervision of a preceptor, the students discuss the proper use of and adherence to the medication, and make recommendations for medication therapy management. The patient meets next with the clinic dietitian; they discuss diet and review basic diabetes information, if necessary. The patient then meets with a primary care provider (either a physician or a nurse practitioner). The primary care provider considers the pharmacy student’s recommendations for medication therapy management as well as those from the other providers and, as needed, initiates new medication therapy, adjusts existing medication therapy, discontinues medications, and orders laboratory tests. Lastly, the patient completes a patient care satisfaction survey assessing the care they received from each team member.

The patient spends about 15 minutes with the nurse, 15 minutes with the pharmacist, 30 minutes with the dietitian, and 30 minutes with the primary care provider. The whole diabetes care team meets at the end of the day to review each case; note recommendations for changes in diet, exercise, lifestyle, medication regimen, and laboratory monitoring; and establish dates for patients to return to the clinic. Pharmacy students provide follow-up telephone calls to each patient the next day to make recommendations and answer any questions.

For this case study, we collected data on 2 measures to provide evidence of the clinic’s benefit to the community: HbA1c values and patient satisfaction. These data continue to be collected. The use of these data for research purposes was approved by the Penobscot Chief on April 3, 2015.

**Clinical measures**. In spring 2013, before the patient’s first clinic visit, data on the patient’s most recent (within 1 year) HbA1c measurement was extracted from the electronic medical record. On the basis of these initial data and guidelines of the American Diabetes Association (9), we classified patients into one of 2 groups: 1) those with HbA1c values ≥7% (uncontrolled diabetes [n = 18]) and 2) those with HbA1c values <7% (controlled diabetes [n = 30]). A follow-up measurement of the same patients’ HbA1c levels was taken in fall 2013 (an average of 250 days after the initial value was recorded). Only patients who attended both the spring and fall clinics were included in these analyses.

**Patient satisfaction and intention to change behaviors.** After each clinic visit, the patient was asked to complete a paper-and-pen survey. Pharmacy students entered the data into SurveyMonkey. Using Likert-scale questions, patients rated their experience overall as well as their experience with each clinic service provider. Patients also rated their likelihood of changing certain diabetes-related behaviors. In addition, the surveys allowed for free-form comments and suggestions for ways to improve the clinic. The diabetes team continues to meet monthly and use this information to improve the clinic’s methods, procedures, and patient flow.

**Statistical analysis.** For the satisfaction surveys, simple descriptive statistics were generated. For HbA1c values, nonparametric testing was performed to account for the small sample size using SPSS (version 21, IBM Corporation). The mean and median values of HbA1c from the baseline measure and follow-up visit were calculated, and the Wilcoxon signed-ranked test was used to detect significant differences between initial values and follow-up values. Regression analysis was used to detect an association between number of clinics attended and change in HbA1c.

## Outcome

The clinic has had successful outcomes. Every month, from February through November, for the past 2 years, a diabetes clinic was held. The pharmacy students (1 to 4 students per 6-week rotation) continue to take primary responsibility for scheduling patients and making reminder telephone calls. This work is appreciated by the health center’s administration and staff. Several students who performed exceptionally well were formally recognized by the tribal chief during 2013 and 2014 with a Penobscot Nation certificate of appreciation. In spring 2015, however, attendance at the clinic waned as some participants dropped out. Diabetes team members met and strategized ways to improve the clinic to better serve the population with diabetes. For example, one suggestion was to dedicate more one-on-one time between patients and various health care providers (at least 2 per visit) and to conduct diabetes-related visits throughout the month instead of focusing on a single clinic day.

**Figure 1:**
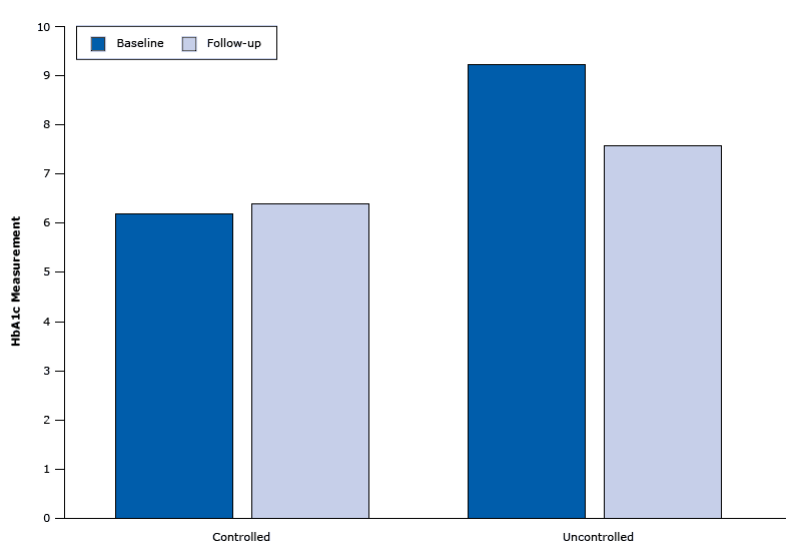
HbA1c measurements at baseline and follow-up among 48 patients in Penobscot Nation Health Center diabetes clinic. Average time between the 2 measures was 250 days. **Table:** 

Among the patients with uncontrolled diabetes, mean HbA1c values declined (but not significantly) from 9.3% at baseline to 8.2% at follow-up, and median HbA1c values declined significantly from 9.3% to 7.6%, (*P* = .004). Among the 30 patients with controlled diabetes at baseline, 25 (83%) had controlled diabetes at follow-up; the median HbA1c in this group increased from 6.2% to 6.4% (Figure 1). When we excluded data on the 5 patients who had controlled diabetes at baseline and uncontrolled diabetes at follow-up, median HbA1c at did not change from baseline to follow-up. An increase in the number of visits per patient was weakly correlated with improved HbA1c levels, but the association was not significant. We had no comparison group. In addition, some patients chose not to return, and those who did return may have been more motivated to comply with recommendations and improve their diabetes control.

Type of diabetes	No. of Participants	Median Baseline HbA1c Measure	Median Follow-Up HbA1c Measure
Uncontrolled	18	9.25	7.6
Controlled	30	6.15	6.4

**Figure 2:**
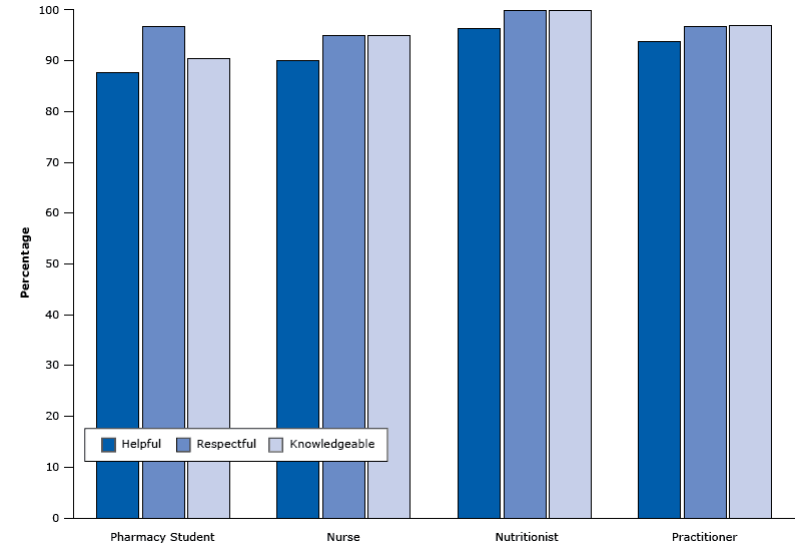
Percentage of survey respondents in the Penobscot Nation Health Center diabetes clinic who agreed (on a 5-point scale) that a provider was helpful, respectful, or knowledgeable. Other points on the scale were somewhat agree, unsure, somewhat disagree, and disagree. **Table:** 

**Figure 3:**
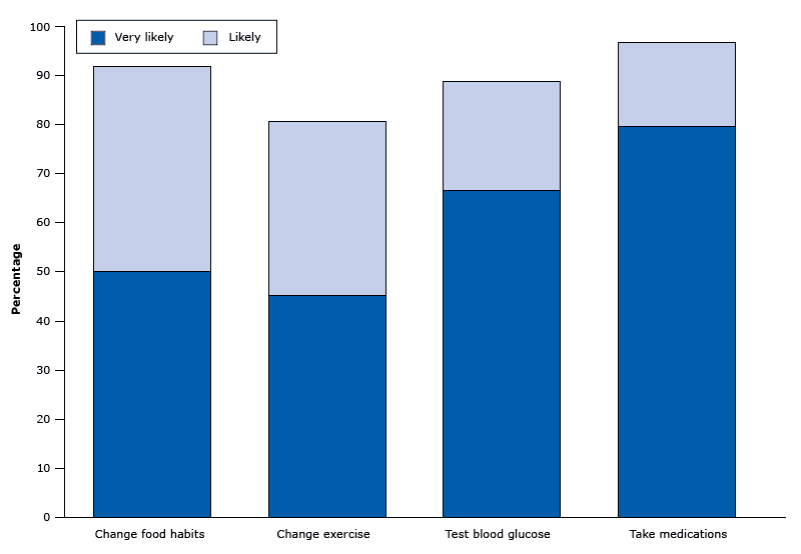
Percentage of survey respondents in the Penobscot Nation Health Center diabetes clinic who indicated they were very likely or likely to change a health behavior. Other points on the scale were not sure, unlikely, and very unlikely. **Table:** 

Satisfaction survey results showed favorable impressions of the clinic in the first year. Participants highly rated all 4 types of providers as helpful, respectful, and knowledgeable (Figure 2). Participants also made positive comments; for example, “[The clinic provides] very good service; [it is] very helpful and encouraging.” Most participants indicated intentions to change diabetes-related behaviors (Figure 3). For example, 96.9% of survey participants said they were very likely or likely to take their medications. Seventy-eight percent of respondents rated the clinic visit as excellent, and 22% of patients rated their visit as good. One patient stated “I thought the Diabetic Clinic was excellent; very informative. I was glad that this was here to be utilized.” Although survey results continue to be favorable, the survey participation rate has declined, and the patients who continue to respond are probably biased. It would be difficult for patients to rate their providers poorly in such a tight-knit community.

Provider Type	Helpful, %	Respectful, %	Knowledgeable, %
Pharmacy student	87.5	96.9	90.6
Nurse	90.0	95.0	95.0
Nutritionist	96.4	100.0	100.0
Practitioner	93.9	96.9	96.9

Health behavior	Very Likely to Change, %	Likely to Change, %
Change food habits	50.0	41.9
Change exercise	45.2	35.5
Test blood glucose	66.7	22.2
Take medications	79.7	17.2

There were and continue to be challenges each month to running a successful diabetes clinic. Occasionally, the primary care provider on the diabetes team is unavailable for a patient visit. When this occurs, the team presents the patient’s medical record to the physician at the end of the clinic day, when decisions are made on any changes to a patient’s care. Frequent cancellations are a common problem for the clinic, as are inconsistent patient telephone numbers, which may explain why many telephone messages go unanswered. In addition, some patients repeatedly decline to be scheduled for a clinic. One reason for this refusal may be the lack of rapport students have with the Penobscot residents compared with established health center staff. Another barrier is a communication breakdown between pharmacy students from rotation to rotation during the 6-week blocks. Continuing to work to build rapport, ensure proficient cultural sensitivity skills, and learn from the experiences of former students may improve the effectiveness of the diabetes clinic.

Another challenge is getting all community members who have been diagnosed with diabetes to visit the clinic. The health center’s staff thinks that one reason for resistance is that community members believe that loss of limbs, blindness, and other complications experienced by their family members are due, or partly due, to the insulin prescribed by providers. To address this issue, pharmacy students work to dispel misunderstandings about insulin among clinic patients. It is hoped that word will spread among community members that these unfortunate outcomes are usually caused by the patient’s long history of uncontrolled diabetes rather than the initiation of insulin therapy. 

An unexpected outcome of the diabetes clinic is the addition of occupational therapy students from the university and a home visitation program, which aims to reach all patients with diabetes who do not visit the clinic. A pilot program began in summer 2015 and consists of home visitation by a local Penobscot pharmacy student, an occupational therapy faculty member, and a student from the university. Patient care and outcome data on this new program were collected and will be analyzed. If the program is successful, the Husson University occupational therapy and pharmacy faculty and students plan to propose an ongoing home care program for patients with diabetes and other chronic debilitating illnesses.

## Interpretation

The partnership of a local pharmacy school, and now an occupational therapy school, and a tribal health center to form an interprofessional diabetes team is on the way to fulfilling the will of the Penobscot people. In its first year, the clinic received high satisfaction ratings from patients, observed a decline of HbA1c levels among patients with uncontrolled diabetes, and expanded health care options for the Penobscot community. The small sample size for measuring changes in HbA1c and the high number of dropouts gives cause for reevaluating and modifying clinic procedures. Discussions among the diabetes team so far have led to the following ideas: reducing the number of diabetes clinic days (making them more “special”), allowing more autonomy for the patients (ie, less structure), adding a financial incentive for program participation, and interviewing patients who have declined participation to determine barriers. In retrospect, we would recommend first interviewing patients with diabetes to seek their input on how best to design the clinic and what services are most needed.

The pharmacy practice site established in the Penobscot Nation Health Center is beneficial for at least some patients with diabetes, the health center, and the pharmacy students. The health center protocol for diabetes care appears to be effective for those who use it. Pharmacy students have a practice site for experiential learning that includes direct care. Continuous quality improvement for the team-based diabetes clinic has been ongoing to streamline and optimize the process for the health center and patients. With further improvements, the clinic is likely to become a strong asset for the Penobscot Nation. An interprofessional team approach to disease management in a close-knit community setting, including students in a health profession, takes time, understanding, relationship building, ongoing procedural problem solving, and long-term commitment by team members.
